# 

**Published:** 1904-08-15

**Authors:** 


### ANNOUNCEMENTS.

PROGRAM OF THE INTERNATIONAL DENTAL FEDERATION.—The Coliseum, St. Louis, Friday, August 26th, at 11:00 A.M. Address of Welcome by Dr. William Conrad, of

St. Louis. Response by Dr. Charles Godon, President. Address by a representative of the Louisiana Purchase Exposition. Address by the Mayor of St. Louis. Response by Dr. H. A. Smith, of Cincinnati, Ohio. Short addresses by the representatives of the foreign countries present, after which the President's Address, Dr. Charles Godon. Report by the Secretary-General, Dr. E. Sauvez. Adjournment, subject to the call of the President.

COMMISSION ON EDUCATION.—Will meet in the Coliseum Building, St. Louis, Mo., August 26th, at 2:00 P.M. Address by the President, Dr. T. W. Brophy, President. A Resume of the Status of Dental Education in France, by Dr. Charles Godon. Some Observations on Technical Education, by Wm. Mitchell, of London, Eng. Address on the Present Status of Dental Education in the United States, by Dr. Gordon White, Nashville, Tennessee. Address on Education, by Dr. R. B. Weiser, of Vienna, Sweden. Drs. H. L. Banzhof, of Milwaukee; C. N. Johnson, of Chicago; M. W. Foster, of Baltimore and W. E. Boardman, of Boston, will also read papers before the Commission.

COMMISSION ON HYGIENE AND PUBLIC DENTAL SERVICE.—Meeting will convene Friday, August 26th, at 2:00 P. M. Address by Prof. W. D. Miller, President, on Dental Hygiene in Germany. Hygiene and Public Service, Dr. E. Forberg, of Stockholm, Sweden. Dr. George Cunningham, of Cambridge, England, will present a report on the Public Dental Service of the late Dr. J. Franck, of Vienna, Austria, with comments.

Other papers will be presented by members of the Commission.

COMMISSION ON INTERNATIONAL DENTAL PRESS.—Meeting will convene Friday, August 26th, at 4:30 P. M. in the French Building at the Fair Grounds. Address by Dr. E. Forberg, President. The Advantage of an International Review, by Dr. A. W. Harlan, New York.

The program for Saturday will be published and distributed early Saturday morning.

Dr. Conrad has secured the use of the French Building for the members of the F. D. I., from 4:00 to 6:00 P. M., Friday, August 26th.

The Executive Council will meet at 6:30 P. M. in the Hotel Jefferson.

Members of the dental profession affiliated with the dental societies of the United States, or in foreign countries, are cordially invited to join any or all the sections of the International Dental Federation.

On behalf of the Federation,

A. W. HARLAN, *Vice-President.*

—✦—

MICHIGAN DENTAL ASSOCIATION OFFICERS.—At the meeting held June 28th and 29th at Lansing, the following officers were elected for the ensuing year: President, Dr. F. H. Essig, of Dowagiac; Vice-President, Dr. John J. Green, of Ionia; Secretary, Dr. A. L. Le Gro, of Three Rivers; Treasurer, Dr. J. Ward House, of Grand Rapids; Clinic Trustee, E. A. Honey, Kalamazoo.

—✦—

TRI-STATE COMMITTEE ON ORGANIZATION.—The following committee was appointed at the meeting of the Michigan State Dental Association to organize the Tri-State Dental meeting for next year: Dr. C. C. Noble, Chairman; Drs. H. C. Raymond, C. H. Worboys, J. J. Green and A. F. Munroe. This committee will have general supervision and make arrangements with the Ohio and Indiana Societies for the meeting, and appoint sub-committees for detailed work.

—✦—

OFFICERS OF HARVARD ALUMNI ASSOCIATION.—President, H. S. Parsons; Vice-President, N. A. Stanley; Secretary, W. E. Boardman, 184 Boylston st., Boston.

American Orthodontists,—A special meeting will be held in the rooms of the Orthodontia Section of the International Congress in the Coliseum, at 10:00 A.M., Monday, August 29th, 1904.

Anna Hopkins, *Secretary.*

—✦—

Mississippi Valley Medical Association.—The thirtieth annual session will be held at Cincinnati, Ohio, October 11th, 12th, 13th, 1904, under the presidency of Dr. Hugh T. Patrick, of Chicago. The headquarters and meeting places will be at the Grand Hotel. The annual orations will be delivered by Dr. Wm. J. Mayo, of Rochester, Minn., in Surgery, and Dr. C. Travis Drennen, of Hot Springs, Ark., in Medicine.

Request for places upon the program, or information in regard to the meeting, can be had by addressing the Secretary, Dr. Henry Enos Tuley, Louisville, Ky., or the Ass't Secretary, Dr. S. C. Stanton, Masonic Temple, Chicago, Ills. The usual railroad rates will be in effect.

H. E. Tuley, *Secretary.*

—✦—

National Dental Association.—At some opportune time during the progress of the Congress, the N. D. A. will meet for the election of officers and the transaction of whatever business may properly come before the association. Very likely some changes to the constitution and by-laws will be considered. Time and place of meeting will be announced at a general session of the Congress.

A. H. Peck, *Secretary.*

—✦—

Journal of Oral Surgery and Medicine.—Dr. A. W. Harlan is to edit a new journal devoted to dental therapeutics under the above title. The first number will appear in January, under the publication management of the Dental Medicine Co., 1122 Broadway, New York. This is a good field, and Dr. Harlan is an experienced editor and capable of making such a journal interesting and of great value to the profession. We welcome the new enterprise and wish it an abundant success.

